# (*E*,*E*)-1,2-Bis[4-(prop-2-yn-1-yl­oxy)benzyl­idene]hydrazine

**DOI:** 10.1107/S160053681104102X

**Published:** 2011-10-08

**Authors:** Wisam Naji Atiyah Al-Mehana, Rosiyah Yahya, Kong Mun Lo

**Affiliations:** a Department of Chemistry, University of Malaya, 50603 Kuala Lumpur, Malaysia

## Abstract

The mol­ecule of the title compound, C_20_H_16_N_2_O_2_, is centrosymmetric with the mid-point of the central N—N bond located on an inversion center. The configuration around the C=N bond is *E*. The whole mol­ecule (except for the H atoms) is approximately planar, with an r.m.s. deviation of 0.07 Å. In the crystal, the presence of weak inter­molecular C—H⋯O hydrogen bonding involving each acetyl­ene H atom and the adjacent phen­oxy O atom results in the formation of supra­molecular chains.

## Related literature

For the structure of (*E,E*)-1,2-bis­[3-meth­oxy-4-(prop-2-yn-1-yl­oxy)benzyl­indene]­hydrazine see: Al-Mehana *et al.* (2011 ▶).
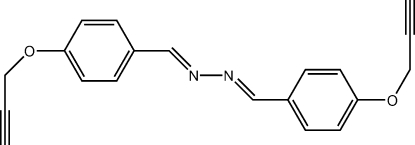


## Experimental

### 

#### Crystal data

C_20_H_16_N_2_O_2_
*M* *_r_* = 316.35
Monoclinic, 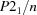

*a* = 7.6598 (1) Å
*b* = 8.1117 (1) Å
*c* = 12.9966 (2) Å
β = 94.466 (1)°
*V* = 805.08 (2) Å^3^
*Z* = 2
Mo *K*α radiation
μ = 0.09 mm^−1^
*T* = 100 K
0.30 × 0.16 × 0.05 mm

#### Data collection

Bruker APEXII CCD area-detector diffractometer
Absorption correction: multi-scan (*SADABS*; Sheldrick, 1996 ▶) *T*_min_ = 0.975, *T*_max_ = 0.996
7404 measured reflections
1847 independent reflections
1698 reflections with *I* > 2σ(*I*)
*R*_int_ = 0.021

#### Refinement

*R*[*F* ^2^ > 2σ(*F* ^2^)] = 0.037
*wR*(*F* ^2^) = 0.111
*S* = 1.01
1847 reflections
113 parameters
H atoms treated by a mixture of independent and constrained refinement
Δρ_max_ = 0.32 e Å^−3^
Δρ_min_ = −0.26 e Å^−3^

### 

Data collection: *APEX2* (Bruker, 2008 ▶); cell refinement: *SAINT* (Bruker, 2008 ▶); data reduction: *SAINT*; program(s) used to solve structure: *SHELXS97* (Sheldrick, 2008 ▶); program(s) used to refine structure: *SHELXL97* (Sheldrick, 2008 ▶); molecular graphics: *X-SEED* (Barbour, 2001 ▶); software used to prepare material for publication: *publCIF* (Westrip, 2010 ▶).

## Supplementary Material

Crystal structure: contains datablock(s) I, global. DOI: 10.1107/S160053681104102X/xu5328sup1.cif

Structure factors: contains datablock(s) I. DOI: 10.1107/S160053681104102X/xu5328Isup2.hkl

Supplementary material file. DOI: 10.1107/S160053681104102X/xu5328Isup3.cml

Additional supplementary materials:  crystallographic information; 3D view; checkCIF report

## Figures and Tables

**Table 1:** Hydrogen-bond geometry (Å, °)

*D*—H⋯*A*	*D*—H	H⋯*A*	*D*⋯*A*	*D*—H⋯*A*
C1—H1⋯O1^i^	0.928 (15)	2.383 (15)	3.2511 (13)	155.7 (13)

Symmetry code: (i) 
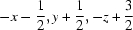
.

**Fig. 1.**
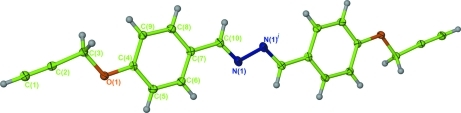
The molecular structure of (E,E)-1,2-bis[4-(prop-2-yn-1-yloxy)benzylindene]hydrazine showing 70% probability displacement ellipsoids and the atom numbering. Hydrogen atoms are drawn as spheres of arbitrary radius.

**Fig. 2.**
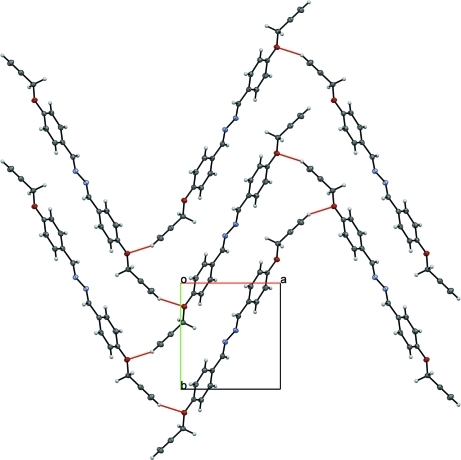
A view of the supramolecular network in the title compound showing the C—H···O interactions.

## supplementary crystallographic information

### Comment

The preceeding study reports the crystal structure of (*E,E*)-1,2-Bis[3-methoxy-4-(prop-2-yn-1-yloxy)benzylindene]hydrazine, in which the molecules are linked by C—H···O interaction between methylene H and methoxy O atoms, resulting in the formation of supramolecular chains (Al-Mehana *et al.* 2011). The title compound, C_20_H_16_N_2_O_2_, without the methoxy substituent on the aromatic ring, is also centrosymmetric around the central azine bond [N1—N1^i^ = 1.412 (2) Å; symmetry operation i: -*x* + 1, -*y* - 1, -*z* + 2]. The molecule also adopts the *E* configuration around the N1=C10 bond [1.2825 (13) Å]. The title compound differs from the previous reported structure as it adopts a different type of C—H···O interaction in its crystal packing. In this case, each acetylene-H atom interacts with the adjacent phenoxy-O [C1—H1···O1^ii^ =3.2511 (13) Å; symmetry operation ii: -0.5 - *x*, 1/2 + *y*, 1.5 - *z*]] resulting in the formation of a supramolecular network (Fig. 2).

### Experimental

4,4'-(*E*, *E*)-hydrazine-1,2-diylidene bis(methan-1-yl-1-ylidene)diphenol (*L*_1_) was prepared by stirring 4-hydroxybenzaldehyde (3 g, 24.5 mmol), hydrazine sulfate (1.65 g, 12.6 mmol) and 1.5 ml of concentrated ammonium solution in a mixture of ethanol and water (20 ml) for 3 h. The product was obtained as a yellow crystalline solid, m.p. 558 K. A mixture of the diphenol, *L*_1_ (2 g, 8.3 mmol) and anhydrous potassium carbonate (1.84 g, 8.6 mmol) in 20 ml of dry acetone was stirred for 20 minutes. Then an excess of propargyl bromide (2.28 g, 19.2 mmol) was added dropwise and the resulting mixture was left under reflux for 48 h. The solvent was then evaporated under reduced pressure. The product was extracted with 100 ml of diethyl ether. The organic layer was washed with brine and dried with MgSO_4_. A yellow amorphous solid was obtained upon slow evaporation of the ethereal solution and was recrystallized with ethyl acetate-methanol mixture to yield the pure yellow crystal, m.p. 453 K.

### Refinement

The acetylene H-atom was located in a difference Fourier map, and was refined isotropically. Other H atoms were placed at calculated positions (C–H 0.93 to 0.98 Å) and were treated as riding on their parent carbon atoms, with *U*(H) set to 1.2 times *U*_eq_(C).

### Crystal data

**Table:**

C_20_H_16_N_2_O_2_	*F*(000) = 332
*M**_r_* = 316.35	*D*_x_ = 1.305 Mg m^−^^3^
Monoclinic, *P*2_1_/*n*	Melting point: 453 K
Hall symbol: -P 2yn	Mo *K*α radiation, λ = 0.71073 Å
*a* = 7.6598 (1) Å	Cell parameters from 4790 reflections
*b* = 8.1117 (1) Å	θ = 3.0–28.3°
*c* = 12.9966 (2) Å	µ = 0.09 mm^−^^1^
β = 94.466 (1)°	*T* = 100 K
*V* = 805.08 (2) Å^3^	Block, yellow
*Z* = 2	0.30 × 0.16 × 0.05 mm

### Data collection

**Table:**

Bruker APEXII CCD area-detector diffractometer	1847 independent reflections
Radiation source: fine-focus sealed tube	1698 reflections with *I* > 2σ(*I*)
graphite	*R*_int_ = 0.021
ω scans	θ_max_ = 27.5°, θ_min_ = 3.0°
Absorption correction: multi-scan (*SADABS*; Sheldrick, 1996)	*h* = −9→9
*T*_min_ = 0.975, *T*_max_ = 0.996	*k* = −10→10
7404 measured reflections	*l* = −16→16

### Refinement

**Table:**

Refinement on *F*^2^	Primary atom site location: structure-invariant direct methods
Least-squares matrix: full	Secondary atom site location: difference Fourier map
*R*[*F*^2^ > 2σ(*F*^2^)] = 0.037	Hydrogen site location: inferred from neighbouring sites
*wR*(*F*^2^) = 0.111	H atoms treated by a mixture of independent and constrained refinement
*S* = 1.01	*w* = 1/[σ^2^(*F*_o_^2^) + (0.0717*P*)^2^ + 0.215*P*] where *P* = (*F*_o_^2^ + 2*F*_c_^2^)/3
1847 reflections	(Δ/σ)_max_ < 0.001
113 parameters	Δρ_max_ = 0.32 e Å^−^^3^
0 restraints	Δρ_min_ = −0.26 e Å^−^^3^

### Special details

**Table:**

Geometry. All e.s.d.'s (except the e.s.d. in the dihedral angle between two l.s. planes) are estimated using the full covariance matrix. The cell e.s.d.'s are taken into account individually in the estimation of e.s.d.'s in distances, angles and torsion angles; correlations between e.s.d.'s in cell parameters are only used when they are defined by crystal symmetry. An approximate (isotropic) treatment of cell e.s.d.'s is used for estimating e.s.d.'s involving l.s. planes.
Refinement. Refinement of *F*^2^ against ALL reflections. The weighted *R*-factor *wR* and goodness of fit *S* are based on *F*^2^, conventional *R*-factors *R* are based on *F*, with *F* set to zero for negative *F*^2^. The threshold expression of *F*^2^ > σ(*F*^2^) is used only for calculating *R*-factors(gt) *etc*. and is not relevant to the choice of reflections for refinement. *R*-factors based on *F*^2^ are statistically about twice as large as those based on *F*, and *R*- factors based on ALL data will be even larger.

### Fractional atomic coordinates and isotropic or equivalent isotropic displacement parameters (Å^2^)

**Table:**

	*x*	*y*	*z*	*U*_iso_*/*U*_eq_	
O1	0.03607 (9)	0.22226 (8)	0.83985 (5)	0.0188 (2)	
N1	0.44709 (10)	−0.44532 (10)	0.96895 (6)	0.0200 (2)	
C1	−0.20330 (14)	0.57486 (13)	0.80143 (8)	0.0249 (2)	
H1	−0.285 (2)	0.6468 (19)	0.7698 (12)	0.040 (4)*	
C2	−0.09922 (13)	0.48192 (12)	0.84201 (7)	0.0198 (2)	
C3	0.02396 (12)	0.37108 (11)	0.89870 (7)	0.0185 (2)	
H3A	0.1381	0.4229	0.9089	0.022*	
H3B	−0.0166	0.3462	0.9658	0.022*	
C4	0.13140 (11)	0.09661 (11)	0.88750 (7)	0.0165 (2)	
C5	0.14991 (13)	−0.04424 (12)	0.82692 (7)	0.0196 (2)	
H5	0.1006	−0.0473	0.7592	0.024*	
C6	0.24152 (12)	−0.17843 (12)	0.86805 (7)	0.0192 (2)	
H6	0.2533	−0.2721	0.8279	0.023*	
C7	0.31723 (12)	−0.17477 (11)	0.97012 (7)	0.0179 (2)	
C8	0.29749 (12)	−0.03295 (12)	1.02859 (7)	0.0186 (2)	
H8	0.3473	−0.0293	1.0961	0.022*	
C9	0.20532 (12)	0.10295 (12)	0.98860 (7)	0.0181 (2)	
H9	0.1932	0.1966	1.0287	0.022*	
C10	0.42043 (12)	−0.31051 (12)	1.01685 (7)	0.0188 (2)	
H10	0.4689	−0.2987	1.0843	0.023*	

### Atomic displacement parameters (Å^2^)

**Table:**

	*U*^11^	*U*^22^	*U*^33^	*U*^12^	*U*^13^	*U*^23^
O1	0.0219 (4)	0.0157 (4)	0.0182 (3)	0.0023 (2)	−0.0021 (3)	−0.0015 (2)
N1	0.0182 (4)	0.0175 (4)	0.0238 (4)	0.0006 (3)	−0.0008 (3)	0.0045 (3)
C1	0.0271 (5)	0.0247 (5)	0.0224 (5)	0.0053 (4)	−0.0016 (4)	−0.0010 (4)
C2	0.0215 (5)	0.0192 (5)	0.0187 (4)	−0.0009 (4)	0.0008 (3)	−0.0028 (3)
C3	0.0198 (5)	0.0161 (4)	0.0191 (4)	0.0004 (3)	−0.0011 (3)	−0.0021 (3)
C4	0.0146 (4)	0.0155 (4)	0.0195 (5)	−0.0007 (3)	0.0014 (3)	0.0018 (3)
C5	0.0216 (5)	0.0197 (5)	0.0173 (4)	−0.0006 (3)	−0.0001 (3)	−0.0008 (3)
C6	0.0211 (5)	0.0159 (4)	0.0207 (5)	−0.0002 (3)	0.0021 (3)	−0.0014 (3)
C7	0.0162 (4)	0.0169 (5)	0.0208 (5)	−0.0013 (3)	0.0028 (3)	0.0028 (3)
C8	0.0175 (4)	0.0204 (5)	0.0176 (4)	−0.0020 (3)	0.0001 (3)	0.0012 (3)
C9	0.0183 (5)	0.0171 (4)	0.0191 (4)	−0.0011 (3)	0.0019 (3)	−0.0017 (3)
C10	0.0166 (4)	0.0192 (5)	0.0205 (4)	−0.0019 (3)	0.0009 (3)	0.0030 (3)

### Geometric parameters (Å, °)

**Table:**

O1—C4	1.3730 (11)	C5—C4	1.4010 (13)
O1—C3	1.4359 (11)	C5—H5	0.9300
N1—C10	1.2825 (13)	C6—H6	0.9300
N1—N1^i^	1.4120 (15)	C7—C8	1.3934 (13)
C1—H1	0.929 (16)	C7—C6	1.4062 (13)
C2—C1	1.1902 (15)	C8—C9	1.3881 (13)
C2—C3	1.4606 (13)	C8—H8	0.9300
C3—H3A	0.9700	C9—H9	0.9300
C3—H3B	0.9700	C10—C7	1.4599 (13)
C4—C9	1.3906 (13)	C10—H10	0.9300
C5—C6	1.3800 (13)		
			
O1—C4—C9	124.18 (8)	C5—C6—H6	119.7
O1—C4—C5	115.18 (8)	C6—C7—C10	123.14 (9)
O1—C3—C2	108.35 (7)	C6—C5—C4	119.75 (9)
O1—C3—H3A	110.0	C6—C5—H5	120.1
O1—C3—H3B	110.0	C7—C6—H6	119.7
N1—C10—C7	122.86 (9)	C7—C10—H10	118.6
N1—C10—H10	118.6	C7—C8—H8	119.2
C1—C2—C3	176.00 (10)	C8—C9—C4	118.83 (9)
C2—C3—H3A	110.0	C8—C9—H9	120.6
C2—C3—H3B	110.0	C8—C7—C6	118.53 (9)
C2—C1—H1	179.5 (10)	C8—C7—C10	118.30 (8)
H3A—C3—H3B	108.4	C9—C4—C5	120.63 (9)
C4—C5—H5	120.1	C9—C8—C7	121.68 (9)
C4—O1—C3	115.98 (7)	C9—C8—H8	119.2
C4—C9—H9	120.6	C10—N1—N1^i^	111.36 (10)
C5—C6—C7	120.57 (9)		
			
O1—C4—C9—C8	179.33 (8)	C5—C4—C9—C8	−0.16 (13)
N1^i^—N1—C10—C7	179.14 (9)	C6—C5—C4—O1	−179.17 (8)
N1—C10—C7—C8	−179.44 (9)	C6—C5—C4—C9	0.36 (14)
N1—C10—C7—C6	−1.46 (15)	C6—C7—C8—C9	0.16 (14)
C1—C2—C3—O1	140.8 (15)	C7—C8—C9—C4	−0.10 (14)
C3—O1—C4—C9	3.86 (13)	C8—C7—C6—C5	0.05 (14)
C3—O1—C4—C5	−176.63 (7)	C10—C7—C8—C9	178.23 (8)
C4—O1—C3—C2	−172.83 (7)	C10—C7—C6—C5	−177.92 (8)
C4—C5—C6—C7	−0.31 (14)		

Symmetry codes: (i) −*x*+1, −*y*−1, −*z*+2.

### Hydrogen-bond geometry (Å, °)

**Table:**

*D*—H···*A*	*D*—H	H···*A*	*D*···*A*	*D*—H···*A*
C1—H1···O1^ii^	0.928 (15)	2.383 (15)	3.2511 (13)	155.7 (13)

Symmetry codes: (ii) −*x*−1/2, *y*+1/2, −*z*+3/2.
